# Non-invasive real-time investigation of colorectal cells tight junctions by Raman microspectroscopy analysis combined with machine learning algorithms for organ-on-chip applications

**DOI:** 10.3389/fbioe.2024.1458404

**Published:** 2024-11-11

**Authors:** A. Calogiuri, D. Bellisario, E. Sciurti, L. Blasi, V. Esposito, F. Casino, P. Siciliano, L. Francioso

**Affiliations:** Institute for Microelectronics and Microsystems IMM-CNR, Via per Monteroni “Campus Ecotekne”, Lecce, Italy

**Keywords:** micro-Raman spectroscopy, machine learning, principal component analysis (PCA), Caco-2 cells, organ-on-chip

## Abstract

**Introduction:**

Colorectal cancer is the third most common malignancy in developed countries. Diagnosis strongly depends on the pathologist’s expertise and laboratory equipment, and patient survival is influenced by the cancer’s stage at detection. Non-invasive spectroscopic techniques can aid early diagnosis, monitor disease progression, and assess changes in physiological parameters in both heterogeneous samples and advanced platforms like Organ-on-Chip (OoC).

**Methods:**

In this study, Raman microspectroscopy combined with Machine Learning was used to analyse structural and biochemical changes in a Caco-2 cell-based intestinal epithelial model before and after treatment with a calcium chelating agent.

**Results:**

The Machine Learning (ML) algorithm successfully classified different epithelium damage conditions, achieving an accuracy of 91.9% using only 7 features. Two data-splitting approaches, “sample-based” and “spectra-based,” were also compared. Further, Raman microspectroscopy results were confirmed by TEER measurements and immunofluorescence staining.

**Discussion:**

Experimental results demonstrate that this approach, combined with supervised Machine Learning, can investigate dynamic biomolecular changes in real-time with high spatial resolution. This represents a promising non-invasive alternative technique for characterizing cells and biological barriers in organoids and OoC platforms, with potential applications in cytology diagnostics, tumor monitoring, and drug efficacy analysis.

## 1 Introduction

Micro-Raman spectroscopy is a technique of great interest that combines Raman spectroscopy with optical microscopy to study the chemical composition of microscopic samples (Mariani et al., 2010). For several years, it has been considered a potential tool for biological and medical applications (Pappas et al., 2000). Raman spectroscopy has several limitations in biological tissue analysis. Its inherently weak signal requires long acquisition times, while tissue fluorescence can mask the spectra, light scattering in tissues reduces spatial resolution and overlapping peaks from various biomolecules complicate compound identification. Additionally, environmental changes, like temperature and humidity, affect spectral reproducibility. Advances in laser technology and detection systems aim to reduce fluorescence interference and improve signal-to-noise ratio while Machine Learning algorithms are being developed for better spectral analysis, expanding the potential of Raman spectroscopy in biological research. Indeed, the importance of this technique lies in its potential to detect biochemical and structural changes in cellular components such as proteins, DNA and lipids by analysing the Raman spectra data associated with the vibrational modes of the molecules and the functional groups of the sample (Mariani et al., 2010; Su et al., 2022). In addition, it allows the study of a wide range of sample sizes, from single cells to intact tissues, in a non-invasive manner without the need for cell/tissue labelling (Pappas et al., 2000). Raman analysis has also the advantage of flexible sampling, allowing measurements on fixed, dried and living cells (Mariani et al., 2010). Recently, this technique has been used, for example, for the identification and possibly grading of lung neoplasia in cell samples (Jess et al., 2009), to classify B-leukemia cells into the different differentiation/maturation stages, and to recognize biochemical changes under chemotherapeutic treatments (Managò et al., 2016), to evaluate cellular modifications after pesticides exposure (Perna et al., 2022); but also for *in vivo* tissue diagnosis of colorectal carcinoma (Fousková et al., 2023), to investigate the resistance mechanisms in a population of cancer cells (Yu et al., 2023) or to identify biochemical changes taking place during the development of Hepatitis C (Ditta et al., 2019). Currently, traditional methods exploited for the study in Organ-on-Chip (OoC) devices allow to evaluate permeability, integrity of cell barriers and their differentiation stage. However, these techniques commonly do not allow working on living epithelia and require preliminary procedures that might influence or change the state of cells in culture. In this context, as a non-destructive, label-free and sensitive technique, Raman microspectroscopy represents a useful investigation tool for OoC devices. OoCs are microfluidic platforms designed to recapitulate the cellular functions and structures in miniature. Due to their ability to mimic the cellular environment, these devices are promising technologies for drug screening and biomedical applications, representing potential substitutes for animal models (Signore et al., 2021; Ingber, 2022). Raman microspectroscopy enables rapid and accurate real-time analysis of biochemical and genetic processes in OoCs by monitoring the different molecular structures of nuclei, lipids and proteins *in situ*, non-destructively and avoiding chip contaminations from electrodes or exotic materials insertion (Zbinden et al., 2020; Tawade and Mastrangeli, 2023).

In this study, the contactless Raman microspectroscopy was used to investigate a live intestinal epithelial model and evaluate alterations of its permeability in a non-invasive way. The intestinal epithelium is an efficient barrier that protects the body from pathogens and toxins and separates the intestinal lumen from the underlying lamina propria (Cherwin et al., 2023). Tight Junctions (TJs) allow intercellular adhesions between epithelial cells and guarantee the integrity of the intestinal barrier (Edelblum and Turner, 2009). Alterations in the transepithelial permeability and modifications of the TJs function have been linked with colorectal cancer and different inflammatory disorders such as inflammatory bowel disease (IBD) (Michielan and D’Incà, 2015). The development of a chip model that mimics the gut, its microenvironment and its functionality represents a useful investigative tool to study permeability alterations and their correlation with specific diseases. Trans-Epithelial Electrical Resistance (TEER) is the most used parameter to verify the TJs formation and to assess the barrier integrity *in vitro*. TEER is a quantitative and non-invasive method based on ohmic resistance calculations or impedance-based measurements (Sciurti et al., 2023). Although the study of TEER can provide several insights into the state of the intestinal epithelium, this technique requires the integration of electrodes into the OoC platform and is influenced by culture parameters such as temperature, medium, cell confluence and user’s skill (Doryab and Schmid, 2022). The aim of our work was to use Raman microspectroscopy as an innovative and non-invasive tool to investigate structural and biochemical alteration of a Caco-2 cells epithelial monolayer. Caco-2 cell line is derived from human colorectal adenocarcinoma and it is a widely used intestinal epithelial model (Lopez-Escalera and Wellejus, 2022). Caco-2 cells form a polarized monolayer over 21 days, with cell-cell adhesions ensured by tight junctions that provide barrier function and low permeability (Liang et al., 2000). Raman microspectroscopy analysis were performed before and after a calcium chelating agent treatment which disrupts TJs and increases epithelial permeability (Orchardt, 1997). Multivariate data analysis tools as Principal Components Analysis (PCA) and a design of supervised machine learning system were used to classify the acquired Raman spectra. This method allowed us to distinguish between intact and different levels of damaged cell junctions in real time, without the need of preliminary procedures (e.g., for immunofluorescent staining), in a rapid, non-invasive, and non-destructive manner. In recent years, Machine Learning (ML) has broadened its applications. In fact, the ability to analyse large quantities of complex data, identify relationships and patterns within datasets, and classify labeled data has driven advancements in spectroscopy analyses. Techniques such as Mass Spectrometry (MS), Near-Infrared (NIR) Spectroscopy, Laser-induced breakdown Spectroscopy (LIBS), Fourier-Transform Infrared (FTIR) Spectroscopy, and Raman Spectroscopy have particularly benefited from these developments, enabling more precise and rapid interpretation of spectral data (Meza Ramirez et al., 2021; Beck et al., 2024; Képeš et al., 2024; Zhou et al., 2024). Our results were correlated with TEER measurements performed in the transwell platforms with live Caco-2 cells and immunofluorescence staining analysis to assess the increase of the epithelial permeability caused by exposure to calcium chelating agent. Raman microspectroscopy coupled with machine learning algorithms is an innovative technique which allows the analysis of dynamic biomolecular variations in real time with high spatial resolution, representing a powerful strategy for biological and medical research.

## 2 Materials and methods

### 2.1 Materials

Caco-2 human colon adenocarcinoma cell lines were purchased from ATCC (Manassas, VA, United States); Dulbecco’s Modified Eagle Medium (DMEM), phosphate buffered saline (PBS, liquid, sterile-filtered, suitable for cell culture), ethylene glycol-bis(2-aminoethylether)- N,N,N′,N′-tetraacetic acid (EGTA) were purchased from Sigma Aldrich (St. Louis, MO, United States). Bovine Serum Albumin (BSA), E-cadherin/CD324 Recombinant Rabbit Monoclonal Antibody, Alexa Fluor 647 (AF647) Goat anti-Rabbit IgG secondary antibody, ZO-1 Mouse Monoclonal Antibody (ZO1-1A12), Alexa Fluor Plus 488 (AF488) Donkey anti-Mouse IgG secondary antibody, Acti-Green Ready Probes 488 Reagent (LifeTechnologies), SlowFade^®^ Gold Antifade Mountant with DAPI were purchased from Thermo Fisher Scientific Inc., Waltham, MA, United States.

### 2.2 Cell line and cell culture

**FIGURE 1:**
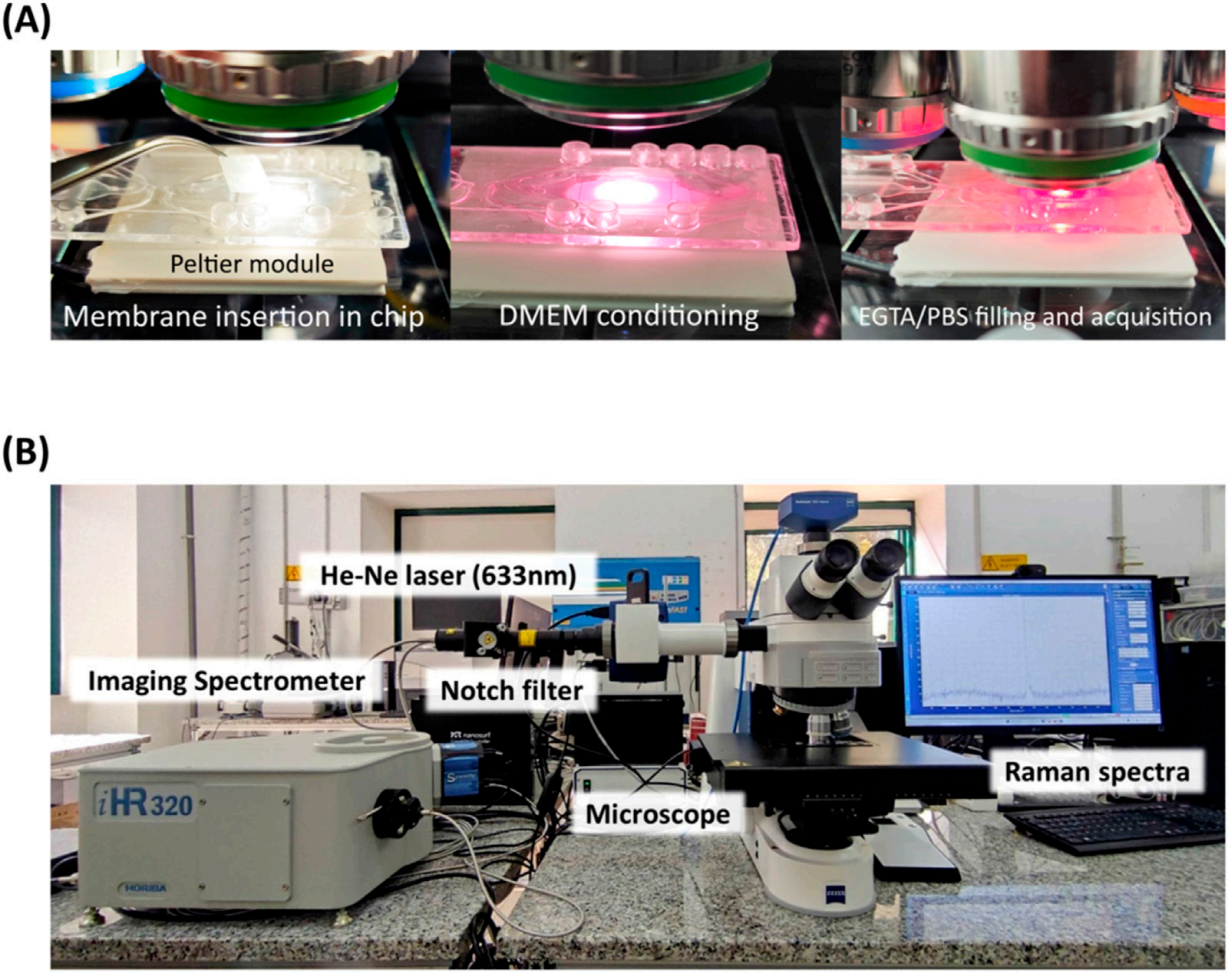
Experimental steps for membrane insertion onto microfluidic chip, equipped with Peltier module temperature control at 37°C **(A)**. Photograph of the Raman microspectroscopy setup **(B)**.

Human epithelial Caco-2 cells were seeded in T-25 cm^2^ flasks and incubated at 37 °C in a humidified atmosphere of 5% CO_2_. The complete culture medium consisted of Minimum Essential Eagle Medium (MEM) supplemented with 10% (v/v) fetal serum bovine (FBS), and 1% (v/v) non-essential amino acid mix solution. At a confluence of 80%–90%, the cells were trypsinized and then seeded at 0.5 × 10^5^ cells per well in 12-well Transwell with 0.4 μm pore polyethylene terephthalate (PET) membrane insert. Caco-2 cells were used after continuous growth for 21 days post-seeding in standard culture conditions to obtain an intact epithelium. Afterwards, the membranes were cut from the transwell insert, rinsed in PBS and placed in a commercial biochip (Fluidic 653 microfluidic ChipShop, Jena, Germany) previously modified with a chamber opening from topside and membrane removal, prior of the insertion of our membranes with epithelium from transwell culture. This step ensures that the PBS does not evaporate during measurements; after the insertion, the chamber was sealed with a custom coverslip and filled with DMEM for conditioning during temperature stabilization and then with PBS/EGTA for Raman analysis (Figure 1A). The temperature control at 37 °C has been realized with a commercial Peltier module connected to a TEC-1091 controller (Meerstetter Engineering, Rubigen, Switzerland).

### 2.3 Raman microspectroscopy

Single - point measurements on Caco-2 cells monolayer were performed using a Raman microspectroscopy apparatus consisting of an imaging spectrometer (iHR320 from Jobin-Yvon Horiba, Sincerity cooled detector) coupled to a Zeiss Axio Imager M2 (Carl Zeiss Microscopy, LLC, White Plains, NY, United States) equipped with a He-Ne laser 633 nm, 17 mW power and 200 μm entrance slit of the spectrometer (Figure 1B).

A diffraction grating with 1800 grooves/mm was used. Raman measurements were conducted with LabSpec 6.7 Spectroscopy Suite Software (Jobin Yvon - Horiba). The spectrometer was calibrated with a silicon wafer before use. Raman spectra were obtained by focusing the laser on selected points of the samples using ×10 and ×20 objectives, in the Raman shift range from 500 to 2000 cm^−1^. Raman analysis were performed first on the cell-free transwell membrane and then on the cells confluent onto the same membrane, before the EGTA treatment and subsequently at 2 and 4 h after the chelating agent exposure. The microfluidic chip was mechanically blocked to the motorized stage in order to guarantee that the mapped acquisition points are exactly the same from experiment start until end of last acquisition, after 4 h of EGTA agent exposure.

### 2.4 Data preprocessing and spectra analysis

Data preprocessing and analysis of acquired spectra was performed using Origin (Pro 2023) software. The Raman spectra underwent the following processing steps: i) cosmic ray removal, this is essential to mitigate the impact of abrupt and isolated peaks introduced into the spectra, which could potentially mask or distort the original spectral features ii) the Savitzky-Golay filter was adopted and applied with a second-order polynomial and a window size of 50 points iii) baseline correction was realized using the Asymmetrical Least Squares Smoothing algorithm, with a smoothing factor 4 in order to reduce external factors like autofluorescence iv) normalization based on the maximum value (Butler et al., 2016; Krafft et al., 2017; Ditta et al., 2019). For each Caco-2 membranes, different Raman spectra were acquired on blank membranes (no cells) and setting the software to acquire a points map over the same intercellular region at 2 h and 4 h of EGTA treatment. The Origin software peak analyzer tool was used to identify the top 20 significant peaks for the intercellular region (corresponding to a TJs abundant location) and the intracellular space from the Raman spectra. The acquired data were analysed by PCA and the spectra were represented along the first 2 principal components vectors in the score plot. The examination of the scores and loading were used to discriminate the different classes of the Raman spectra (Frausto-Reyes et al., 2005). To detect and classify different conditions of the Caco-2 monolayer using Raman spectra measurements, a supervised machine learning system was designed and trained with labeled measurements on Matlab2021 software. The detection and classification problems were solved by classifying the Raman measurements into one of the four available classes (t = 0, t = 2 h, t = 4 h, empty/blank membrane). The most important peaks for a correct classification of the four classes were identified through analysis of loadings related to the first most significant 9 principal components (explaining 99% of the total variance). For each principal component, the 30 loadings with the highest absolute value were extracted. The peaks associated with all the heaviest PC loadings were then extracted for each acquired measurement and for all conditions. The extracted features were ranked by importance coefficients extracted by the training of a linear SVM algorithm and then subsequently selected to obtain the optimal number of features that maximize the performance of the model (Guo et al., 2002; Kim and Rattakorn, 2011; Rebrošová et al., 2017). For each class, the dataframe of labeled features was randomly divided in two subsets (Lee et al., 2024): 80% of the dataset was used as a training set and 20% of the dataset was used as a testing set. To avoid overfitting, the presented classification approaches were performed using 10-fold cross-validation for the validation step. In this method, the training data was first divided into k-folds of equal size. Then, k-1 fold was used to test the classifier, and the remaining folds were used for training. This process was repeated k times, with each fold being used exactly once as the test set (Kohavi, 1995). This method guarantees that every peak set in the dataset was used for both training and testing. The training accuracies were subsequently calculated as average on the remaining 10% of the training data that was held out for each testing.

### 2.5 TEER measurements

To monitor the growth of the Caco-2 cells, to assess the formation of tight junctions (TJs) and to test the effect of EGTA on the integrity of the Caco-2 monolayer, TEER measurements were performed in Transwell using impedance spectroscopy technique. Chopstick-like parallel electrodes were custom made with 2 tungsten needles, 800 µm diameter, inserter in a PTFE circular disk at 10 mm gap, in order to allow insertion of the needles in the apical and basolateral compartments of the transwell insert in a reliable way. Needles length was regulated to avoid mechanical contact with multiwell bottom and with the Transwell membrane. Impedance spectra were recorded over a frequency range of 1 Hz–100 kHz at an alternating potential of 10 mV, using a potentiostat/galvanostat (PalmSens EmStat 4 M model). Impedance measurements were performed for 32 days, before and after the EGTA treatment and the acquired data were fitted with an optimized equivalent electric circuit using Zview software (Scribner Associates Inc., Southern Pines, NC, United States) to obtain TEER values.

### 2.6 Immunofluorescent staining

Caco-2 cells were rinsed with PBS and fixed with 4% paraformaldehyde for 15 min, at room temperature (RT). F-actin was labeled by following the manufacture’s protocol using Acti-Green Ready Probes 488 Reagent (LifeTechnologies). Briefly, after permeabilization with 0.1% (v/v) Triton X-100, cells were incubated with few drops of staining solution for 30 min in the dark at RT and then rinsed three times in PBS. Caco-2 cells were labeled with E-cadherin Monoclonal Antibody at a dilution of 1:500 in PBS with 1% BSA and incubated for 3 h at RT. After three washes, cells were incubated with AF647 secondary antibody at a dilution of 1:400 for 30 min at RT. Tight junction protein ZO-1 staining was carried out by incubating Caco-2 cells with ZO-1 Monoclonal Antibody at a dilution of 1:100 in 0.1% BSA, overnight at 4 °C. After three washes, cells were incubated with AF488 secondary antibody at a dilution of 1:2000 for 45 min at RT. Lastly, nuclei were stained with SlowFade^®^ Gold Antifade Mountant with DAPI and imaged with Zeiss Axio Imager M2.

## 3 Results and discussion

### 3.1 Raman analysis on fixed cells

To optimize our experimental setup in terms of calibration and acquisition parameters of the Raman analysis protocol for the cellular compartment characterizations, a series of preliminary analyses were performed. First, an intact cell monolayer (21 days after seeding) on the transwell membrane was fixed with 4% paraformaldehyde. Using the LabSpec 6.7 Spectroscopy Suite software, 10 spectra in the intracellular space and 10 spectra in the intercellular space were selected from the live image acquired with a ×20 objective. By focusing the laser beam at the selected points, Raman spectra of the different cellular compartments were recorded and analysed. A prior review of the literature (Talari et al., 2015; Brozek-Pluska, 2020; Zhang et al., 2020; Pezzotti, 2021; Beton-Mysur and Brozek-Pluska, 2023), summarized in Table 1, was essential to identify the molecular functional groups and bonds structure vs. detected peaks.

**TABLE 1:** The assignments of Raman peaks for characterization of Caco-2 and eukaryotic cell (Talari et al., 2015; Brozek-Pluska, 2020; Zhang et al., 2020; Pezzotti, 2021; Beton-Mysur and Brozek-Pluska, 2023).

Peak no.	Center (cm^−1^)	Major assignment
1	621 cm^−1^	Phenylalanine
2	667–669 cm^−1^	Cystine (collagen type I), Nucleic acids T and G
3	671 cm^−1^	Nucleic acids T and G
4	678 cm^−1^	Ring breathing modes in the DNA bases
5	729 cm^−1^	Nucleic acids A
6	742 cm^−1^	DNA, tryptophan
7	815 cm^−1^	Proline, hydroxyproline, tyrosine, PO2- stretch
8	852–858 cm^−1^	Proline, hydroxyproline, tyrosine
9	860 cm^−1^	Phosphate group
10	893 cm^−1^	Phosphodiester, deoxyribose
11	920 cm^−1^	Proline ring, glucose, lactic acid and praline ring
12	928 cm^−1^	Proline and Valine
13	937–938 cm^−1^	Proline, hydroxyproline
14	1,003 cm^−1^	Phenylalanine
15	1,035 cm^−1^	Collagen
16	1,053 cm^−1^	C-O stretching, C-N stretching (proteins)
17	1,080 cm^−1^	sE-cadherine
18	1,090 cm^−1^	PO_2_- stretch
19	1,230 cm^−1^	Amide III
20	1,298 cm^−1^	Fatty acids, CH_2_ deformation
21	1,614 cm^−1^	Tyrosine

**FIGURE 2:**
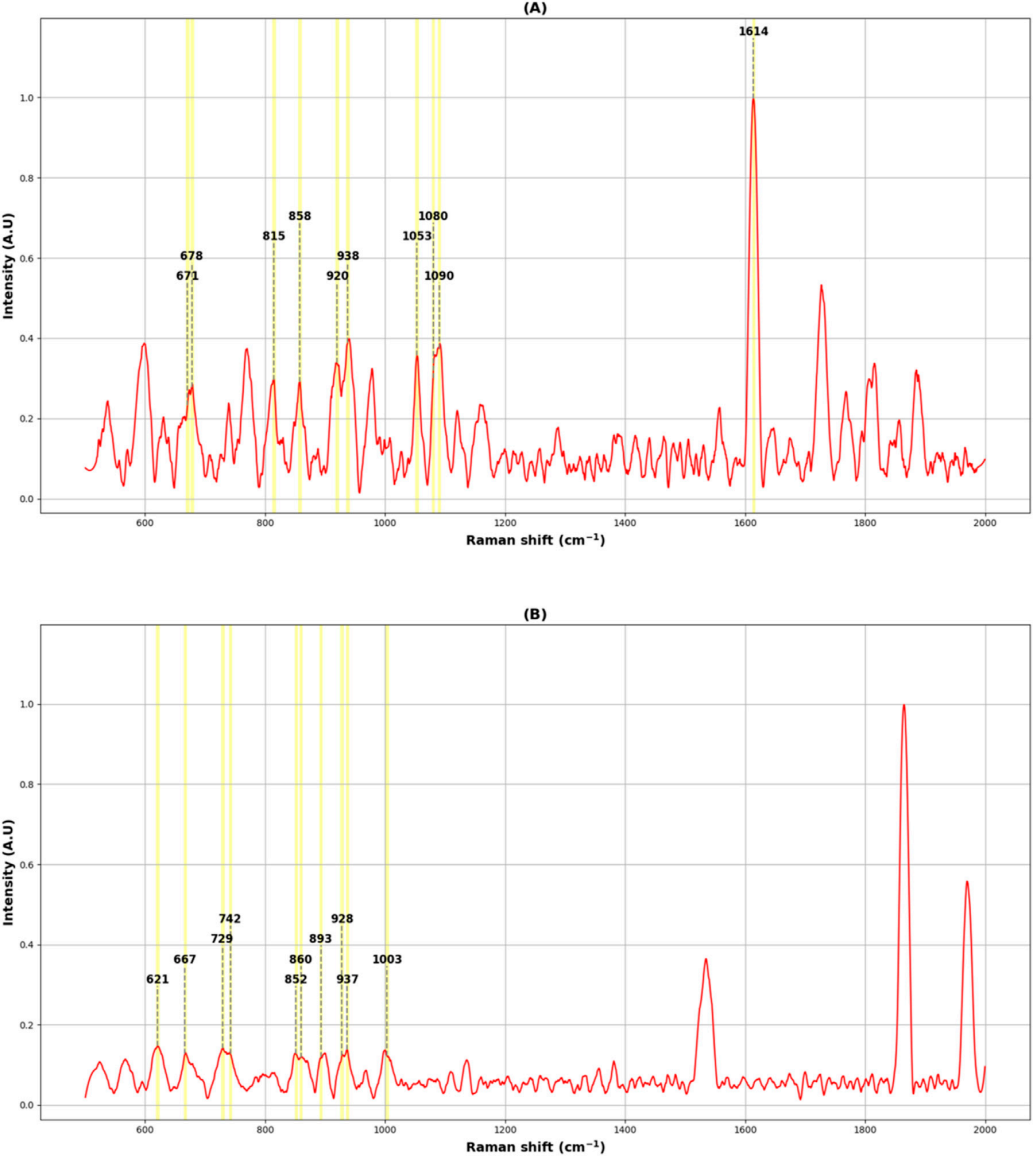
Average Raman spectra of the Caco-2 monolayer acquired from the intercellular region **(A)** and the intracellular region **(B)**. The identified peaks associated with biological molecules in the TJ area were: 671 cm^−1^, 678 cm^−1^, 815 cm^−1^, 858 cm^−1^, 920 cm^−1^, 938 cm^−1^, 1,053 cm^−1^,1080 cm^−1^, 1,090 cm^−1^ and 1,614 cm^−1^; for the intracellular space, the identified peaks were: 621 cm^−1^, 667 cm^−1^, 729 cm^−1^, 742 cm^−1^, 852 cm^−1^, 860 cm^−1^, 893 cm^−1^, 928 cm^−1^, 937 cm^−1^and 1,003 cm^−1^.

A comparison between the Raman signals detected in our analyses and those reported in the literature, allowed us to identify peaks uniquely associated with the intracellular compartment or the cell membranes (intercellular) region. As shown in Figures 2A, B, the most significant peaks found by averaging the spectra extracted from the intracellular region were at 621 cm^−1^, 667 cm^−1^, 729 cm^−1^, 742 cm^−1^, 852 cm^−1^, 860 cm^−1^, 893 cm^−1^, 928 cm^−1^, 937 cm^−1^ and 1,003 cm^−1^. From cell membrane spectra, intense peaks were identified at 1,614 cm^−1^, 671 cm^−1^, 678 cm^−1^, 815 cm^−1^, 858 cm^−1^, 920 cm^−1^, 938 cm^−1^, 1,053 cm^−1^, 1,090 cm^−1^ related to different molecular structures (Table 1) and a significant band at 1,080 cm^−1^ was associated with E-cadherin, one of the adhesion proteins involved in the cell-cell interactions.

**FIGURE 3:**
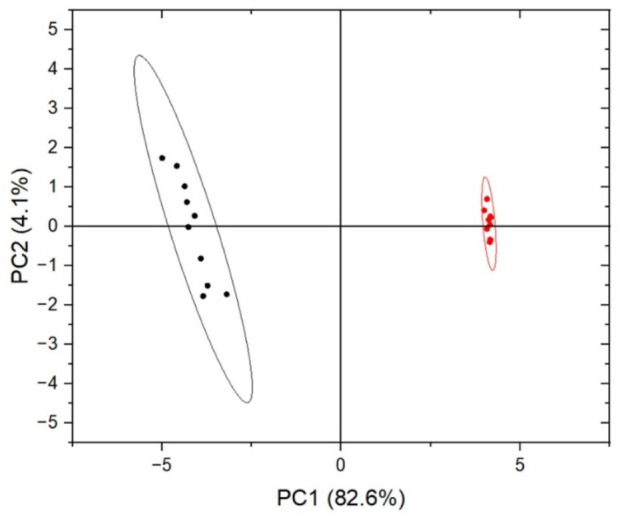
Score plot of PCA on fixed cells experiment. Each Raman spectrum was represented by dots (red for the intracellular region and black for the cellular membrane), transformed in the two principal eigenvectors, PC1 (82.6%) and PC2 (4.1%) space.

A depth exploration of the Raman spectra was also performed using PCA method. The data were clearly separated into two clusters corresponding to different cellular compartments, with an explained total variance of 86.7% on the first two principal components (82.6% on PC1 and 4.1% on PC2), indicating a strong differentiation between the two analysed cellular regions. The reported results in Figure 2 demonstrate that Raman microspectroscopy allowed us to observe the different contributions of biological components composing the Raman spectrum, reflecting the variability of biochemical constitution in the analysed cellular compartments (Table 1). Additionally, Figure 3 shows that Raman microspectroscopy can distinguish different compartments of fixed epithelial cells by PCA (Shin et al., 2018).

Starting for this preliminary assessment, the experimental study will focus on the TJs regions, acquiring multiple spectra with the laser spot focused on the cell-cell junctions areas, subsequently processed.

### 3.2 Raman microspectroscopy on live cells

To evaluate the performance of Raman microspectroscopy on live cells as a potential tool for the non-invasive analysis of the state of the intestinal epithelium in an OoC system, live Caco-2 cells were examined. The permeability of the Caco-2 monolayer, as an intestinal model, and the integrity of TJs were investigated by comparing the cell status 21 days after seeding and 2 and 4 h after EGTA exposure. EGTA, through the extracellular depletion of Ca^2+^, induces the disruption of the TJs and the formation of epithelial hot spots, leading to a gradual decrease in the epithelial barrier functions (Panou et al., 2023).

**FIGURE 4:**
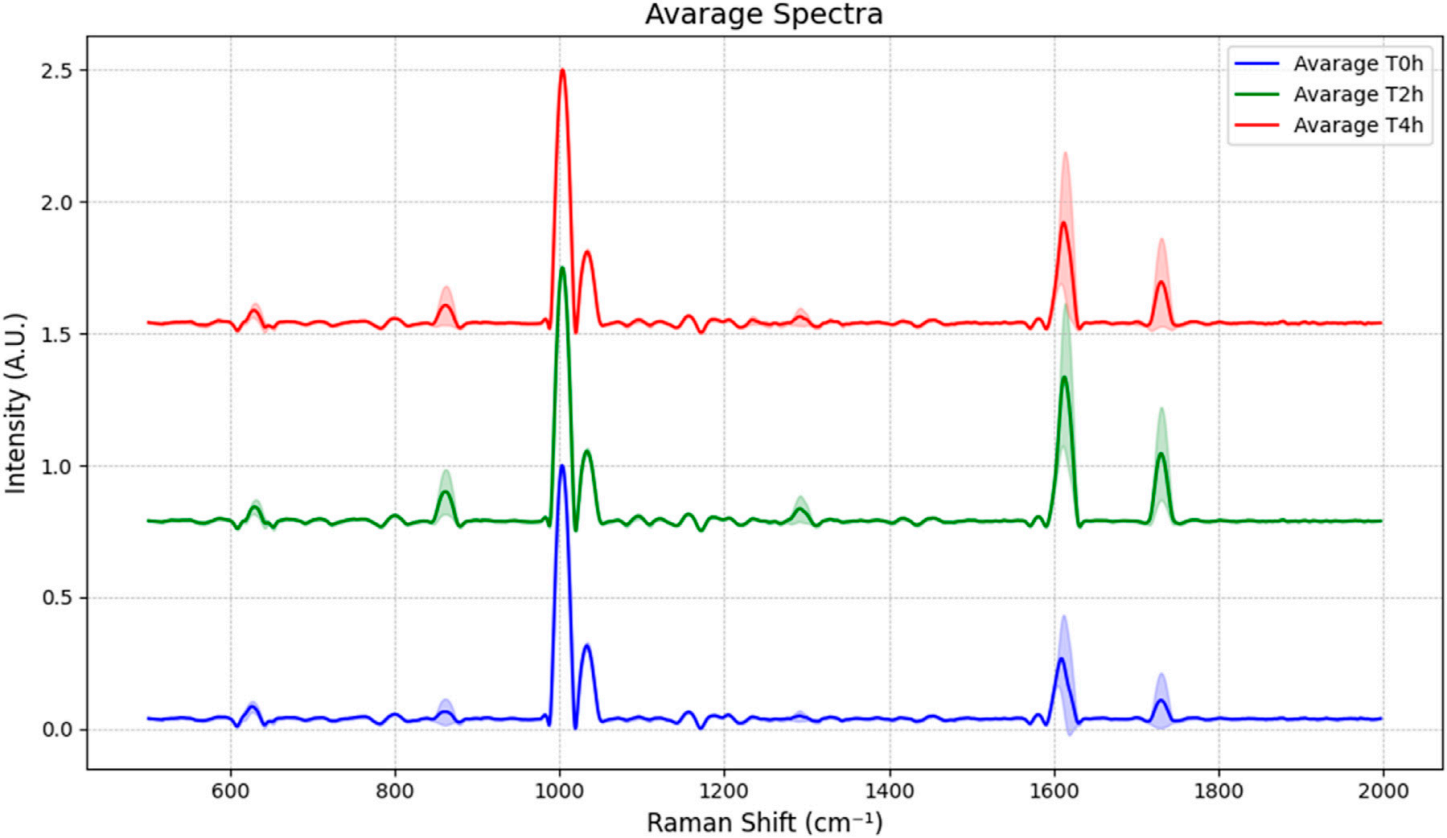
Average spectra of the live cells samples at T = 0 h, after 2 h and 4 h of exposure to 5 mM EGTA. The shaded area surrounding each peak represents the standard deviation.

To ensure that the epithelium analysed by Raman spectroscopy exhibits alterations similar to those observed in samples analysed exclusively by TEER and IF after EGTA treatment, the cell culture was maintained until the characteristic permeability of fully intestinal differentiated cells was achieved in our experimental system. The PET transparent membrane of the Transwell insert on which the cells were cultured was cut and immersed in 5 mM EGTA solution in the microfluidic chip reported above. Raman spectra were acquired using three different batches and from each, 20 selected spots distributed in the intercellular space at different time points of EGTA exposure (time 0, 2 h, 4 h). The recorded data were pre-processed as reported in section 2.4 and the average spectra at different treatment conditions are shown in Figure 4.

**FIGURE 5:**
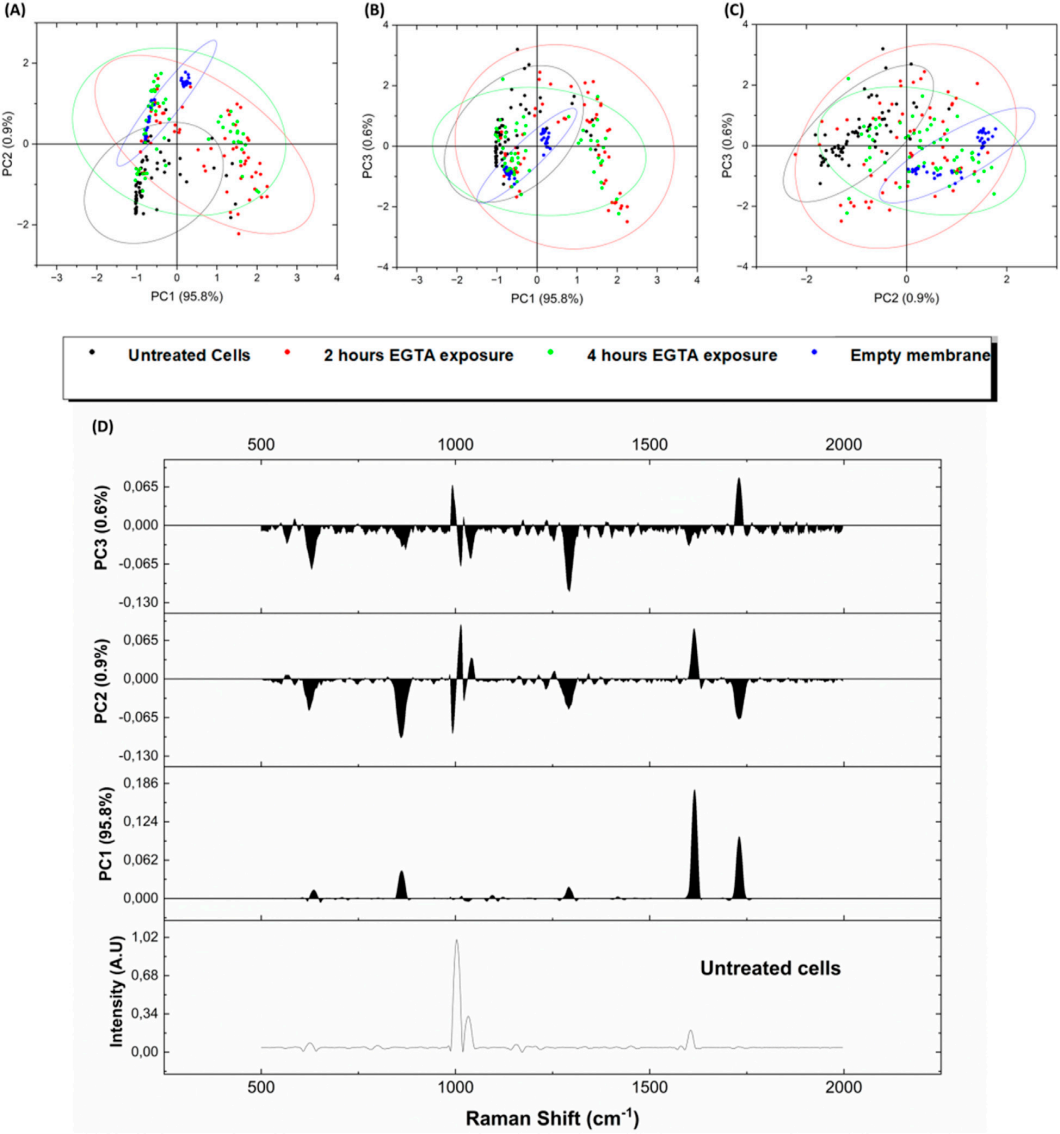
Score Plots of PCA performed on whole acquired spectra from live cells samples with different EGTA exposures **(A)** PC1 vs. PC2, **(B)** PC1 vs. PC3 and **(C)** PC2 vs. PC3. Black dots = untreated cells (T0), red dots = 2 h EGTA exposure (T2), green dots = 4 h EGTA exposure (T4), blue dots = cell-free membranes (blank). **(D)** Loading plots for the three first principal components PC1, PC2 and PC3 and correlation of loading weights vs. the reference spectra of intact epithelium (T0-no EGTA treatment).

The Raman spectra from the intercellular space were then analysed by PCA in order to simplify the complexity of acquired spectral data by using a lower number of unrelated variables, the principal components (PCs). As shown in Figures 5A, B, C the high explained variance of the first three principal components, adding up to 97.3% (PC1 at 95.2%, PC2 at 0.9%, and PC3 at 0.6%), confirms that the first graph of PC1 vs. PC2 is the projection that better reports a good discrimination between the untreated epithelium and the blank membrane (no cells), while the points related to 2 h and 4 h EGTA treatment are mainly superposed, indicating that the 2 h treatment caused a detectable damage (not so different from 4 h spectra) of the TJ as confirmed also by the immunofluorescence investigation results reported in section 3.4.

Figure 5D reports the loading plots for the three first principal components PC1, PC2 and PC3 and correlation of loading weights vs. the reference spectra of intact epithelium (Untreated cells spectrum in Figure 5D). The underlying spectral informative data can be explained, e.g., on the PC1 component by the corresponding increased proteins-related features in the 852–858 cm^−1^ region (proline, hydroxyproline) and 1,614 cm^−1^ shift, related to aromatic band (Tyrosine, Tryptophan, Phenylalanine) (Talari et al., 2015; Beton-Mysur and Brozek-Pluska, 2023).

In order to generate a classification tool able to distinguish intact from damaged junctions, the Raman data were used to train a supervised classification algorithm using only the most determinant peaks (Ressom et al., 2007). From the previous PCA, the loadings were used to extract specific peaks and then used as predictors for the training of a supervised ML classification model. The intensity of peaks with the highest absolute loadings were selected (see section 2.4) from the loadings matrix of the first 9 principal components (which together explain 99% of the variance in the data), identifying peaks that can capture the difference between conditions, ensuring that our model is trained on the most discriminative features of the data (King and Jackson, 1999; Li et al., 2016). The total data set of 220 analysed spectra included 60 spectra for each status of the Caco-2 monolayer (time 0 h, 2 h, 4 h) acquired from three similar samples and 40 spectra related to empty membrane areas. After extraction and elimination of duplicate peaks, a total of 162 predictors were found. They were then ranked according to the feature importance score (Brank et al., 2002; Wang et al., 2022) (calculated from SVM weights) obtained from outputs of the learning process of an SVM model with a “linear” kernel, box constraint of 10, and with “one vs. all” options, trained with the feature extracted by all measures (Brank et al., 2002; Wang et al., 2022). To train and evaluate the ML model, the dataset was randomly divided into training sets (80%) and test sets (20%) [“spectra method” splitting (Guo et al., 2017)]. The best ML classifiers were selected using the Classification Learner application of Matlab 2021. Table 2 summarizes the main hyperparameters of the ML models used in this work. The information about the performance of the classification ML model is typically summarized into a confusion matrix from which a fundamental parameter, the accuracy (Acc), can be calculated. To avoid overfitting, the classification was performed using a 10-fold cross-validation scheme and different classification predictive algorithms were compared. The number of features was progressively reduced until the optimal set was identified, where further addition of features did not improve the model’s performance or led to a decrease in accuracy (Chemmakha et al., 2022). With an optimal number of 13 features, a robust performance was achieved in both validation and test phase results.

**TABLE 2:** Main hyperparameters of the ML models used in this work.

Model	Hyperparameter	Value
Quadratic SVM	Kernel Function	Quadratic
	Kernel Scale	Automatic
	Box constraint level	1
	Multiclass method	One-vs-One
	Standardize Data	Yes
Cubic SVM	Kernel Function	Cubic
	Kernel Scale	Automatic
	Box constraint level	1
	Multiclass method	One-vs-One
	Standardize Data	Yes
Wide Neural Network	Number of fully connected layer	1
	First layer size	100
	Activation	ReLU
	Iteration limit	1,000
	Regularization Strenght	0
	Standardize Data	Yes

Table 3 compares the performance of different classification models, Quadratic SVM (qSVM), Cubic SVM (cSVM) and Wide Neural Networks (WNN) through the quantitative metrics, Specificity, Precision, Sensitivity, F1 Score on the testing data and Accuracy, which was also calculated for the training.

**TABLE 3:** Performance metrics (Train Accuracy, Test Accuracy, Specificity, Precision, Sensitivity (or Recall), F1 Score).

	Train accuracy	Test accuracy	Specificity	Precision	Sensitivity	F1 score
Quadratic SVM	0.903	0.977	0.992	0.981	0.979	0.977
Cubic SVM	0.909	0.909	0.969	0.904	0.917	0.909
Wide Neural Networks	0.920	0.886	0.958	0.901	0.875	0.886

**FIGURE 6:**
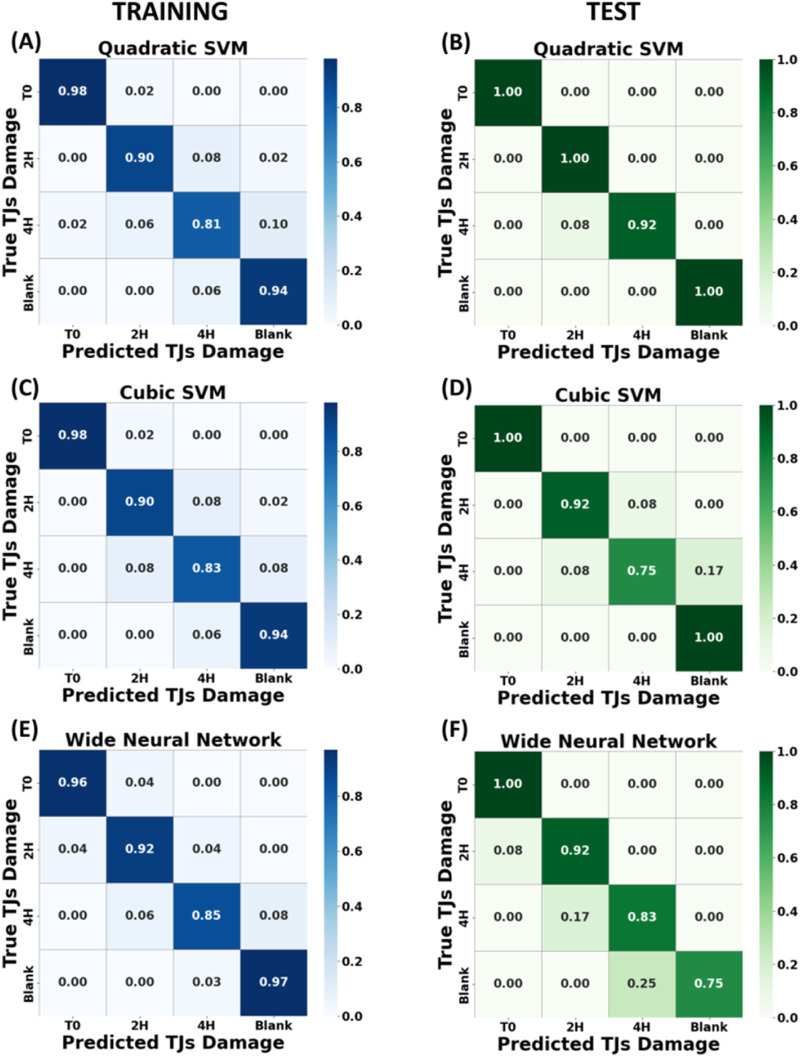
Normalized confusion matrix across four classes of algorithms trained with dataset split by “spectra method” and corresponding to different EGTA exposure times (T0, T2 h, T4 h and empty membrane). In the left panels are illustrated the confusion matrices of the training phase for Quadratic SVM **(A)**, Cubic SVM **(C)** and Wide Neural Network **(E)**”. In the right panels, the corresponding accuracy confusion matrices for test phase **(B)**, **(D)** and **(F)**.

As shown in Figure 6 after the validation phase, high accuracy values were obtained for all analysed models, and very promising results were confirmed in the testing phase. The validation accuracies for the qSVM, cSVM, the Neural Network model with WNN were 90.7%, 90.9% and 92.0% respectively, and the downstream test phase accuracies were 97.2%, 90.9%, and 88.6%, respectively. These results demonstrate the ability of the presented models to discriminate different levels of epithelium damage and their generalization ability. A slight misclassification was observed for training and test phases mainly between classes 2 h and 4 h treatments; this output was correlated to a fast degradation of tight junctions after 2 h treatment, as explained and supported by the immunostaining fluorescence tests in paragraph 3.4.

To evaluate the reason behind the high performance of the previously obtained models, a focus was placed on the nature of the features that enables the accurate identification of the epithelium state. All the 13 Raman shifts with their associated intensities were assigned in several biological related peaks, listed in Table 1, including 621, 1,003, 1,035, 1,230, 1,298, 1,614 cm-1. These differences in the intercellular space fingerprint are attributable to structural and compositional changes of proteins and fatty acids that were induced by the treatment. Not all features coincide with the peak centre of the biological components, but rather they are located in large peaks shoulder where there is a major difference between classes (Yang et al., 2024).

Since the Raman measurements were conducted on live tissues grown on the same porous membrane and presenting comparable TEER values, this study established a correlation between the predicted damage status and the TEER measurements recorded at the corresponding treatments with EGTA. As reported in the manuscript in paragraph 3.4, the measured TEER values are 700 ± 26 Ω‧cm^2^ of the intact epithelium, 328.9 ± 9.1 Ω‧cm^2^ after 2 h and 197.9 ± 4.5 Ω‧cm^2^ at 4 h of EGTA treatment; these values are related to classes T0, T2 and T4 classified by the ML algorithm, respectively.

### 3.3 Real scenario damage assessment by optimized ML models on never seen live epithelium

In clinical applications, measurements acquired from different biological samples may exhibit consistent results variance, so different methods of dataset splitting can have a large impact on the accuracy of the test and may result in overly optimistic performance estimates (Wu et al., 2021; Blake et al., 2022). In order to reduce the effect of the variance of the data set and to further investigate the applicability of the model in a clinical setting, the splitting of dataset in training set and test set at the highest hierarchical level, is strongly recommended (Guo et al., 2017). Present work adopted this suggested splitting approach, reporting the identification results below. In our case, the different membranes from which measurements have been acquired, namely, “sample-based” (Guo et al., 2017), was considered the highest hierarchical level. To develop a tool that can correctly predict the Raman spectra even into a real clinical scenario application (where algorithm must be applied to unknow tissue samples), the training of the ML classification algorithm was performed using the highest hierarchical level splitting of the dataset, as explained above. The training and the validation of the models were carried out by using only a restricted number of samples, in our case 160 labelled spectra from all damage cases, acquired from two of the three membranes. From the last membrane, 60 spectra acquired from an unknown treated epithelium observed for the first time were used to test the capability of the model to identify the right and effective damage level of the live epithelium. This protocol simulates the application of our developed algorithm applied for the first time to a patient biopsy or to an OoC device with optical laser access on cells area for this non-invasive analysis. In this case, the peaks with the highest absolute loading were selected from the loading matrix of the first five principal components (99% of the variance) extracted by the PCA performed on the spectra obtained from two of the three previous membranes (160 spectra), identifying 97 predictors (peaks). The predictors were ranked again according to the feature importance score as reported in the previous paragraph.

**FIGURE 7:**
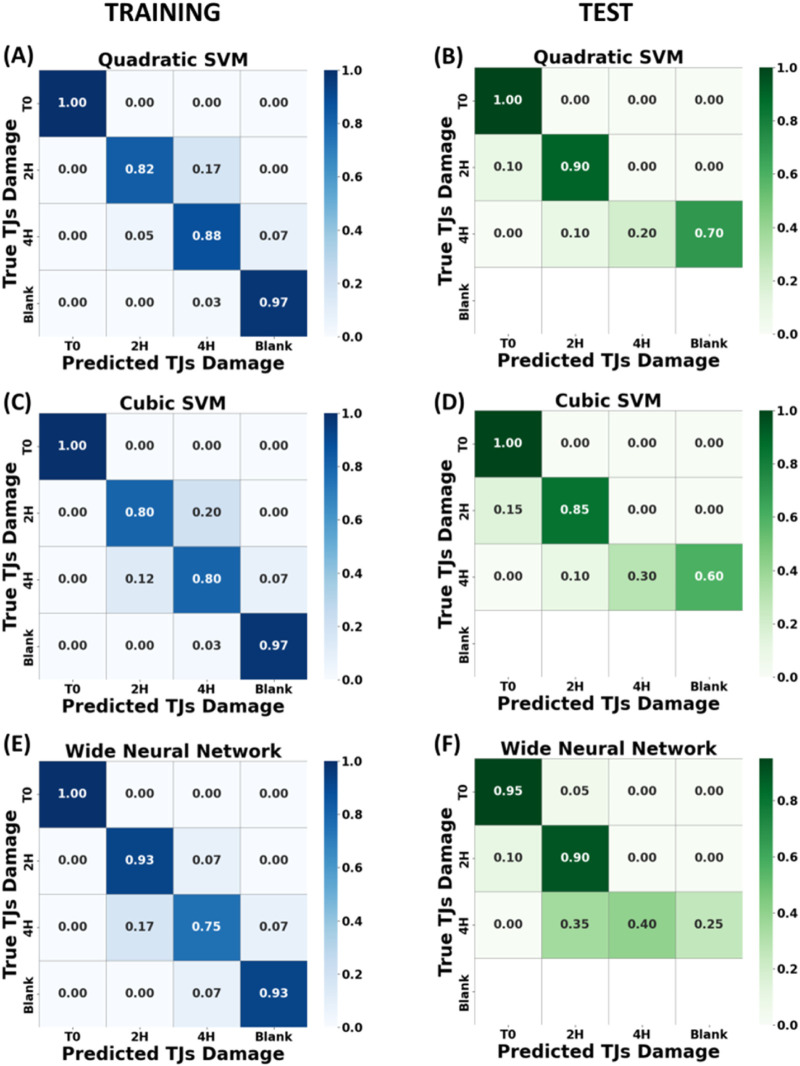
Heatmap of the cross-validation accuracy across four classes, corresponding to different EGTA exposure times (T0, T2 h, T4 h and blank membrane), trained with dataset split by “sample-based” method. In the left panels are illustrated the confusion matrices for the training phase for Quadratic SVM **(A)**, Cubic SVM **(C)** and Wide Neural Network **(E)** algorithms. In the right panels, the corresponding accuracy confusion matrices for test phase **(B)**, **(D)** and **(F)**. The fourth row on the test confusion matrix is blank because the test measurements were performed on intact, 2 h and 4 h treated epithelia only.

As shown in Figure 7 promising results were obtained after the validation step; all selected models were able to discriminate between intact/untreated (Class 0) vs. damaged junctions (Class 1 and 2) and between minor junction damage and severe damage (Class 1 vs Class 2), in both the validation and testing phases. The fourth row on the test confusion matrix is blank because the test measurements were performed on intact, 2 h and 4 h treated epithelia only, missing the blank substrate as in a real clinical test (no interest to investigate cell-free chips or blank supports). The best model training result was performed by qSVM (Figure 7A), reaching an accuracy of 91.9% with 7 peaks as optimal features number, with the optimal set identified using the same method described in Section 3.2. Comparable training results were also obtained by the cSVM (Figure 7C) and the WNN algorithm (Figure 7E), with a validation accuracy of 89.4%, respectively. Despite the good performance in the validation step, different outcomes show up in the testing step, as shown in Figures 7B, D and F with an accuracy of 70% for the quadratic SVM model, 71.7% for the cubic SVM model and 75% for the WNN model. Indeed, all the models suffer in discriminating between heavily damaged epithelium and cell-free membranes (Class 2 vs. Class 3) due to the physical and biological similarity between these two epithelia, because of the significant injury induced by a 4 h EGTA treatment and observed detachment of the cells (see Figure 9 fluorescence data for details). In addition, the 7 Raman shifts features exploited to differentiate the acquisitions into the 4 classes align with the peaks of specific biomolecules identified in previous analyses. These biomolecules include Phenylalanine (observed at 621 cm⁻^1^ and 1,003 cm⁻^1^), phosphate groups (at 860 cm⁻^1^), and fatty acids (at 1,298 cm⁻^1^). This suggests that the spectral changes detected by the ML model after EGTA treatment occur in regions associated with these key biological components of the cell membrane, highlighting the congruence between the observed spectral variations and the molecular composition of the membrane. In conclusion, it is well known [39] that “sample-based” splitting method of dataset leads to a decrease in model performance compared to the standard “by spectra-based” method. Nevertheless, it represents a more realistic method to assess how well the model would perform in the real application setting and better understand the influence of the data heterogeneity.

### 3.4 TEER and immunostaining-supported validation of the ML algorithms

**FIGURE 8:**
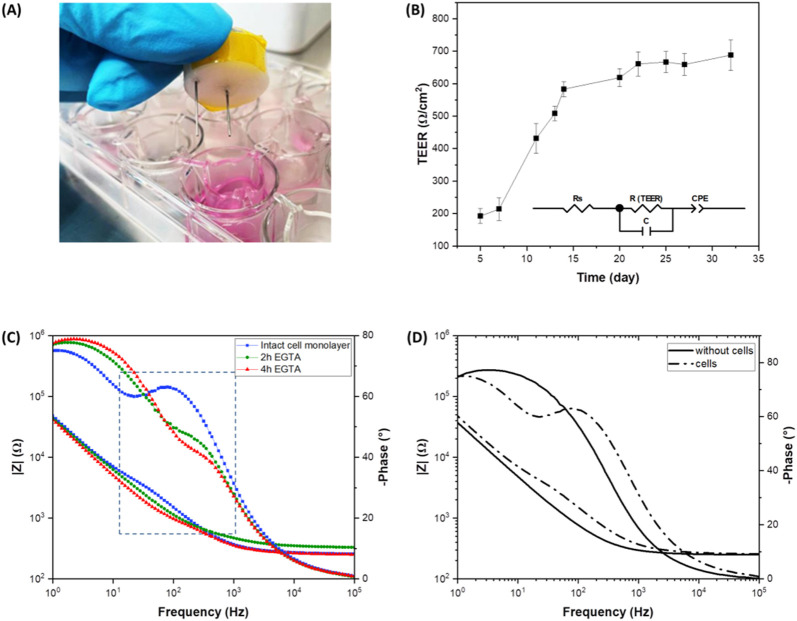
Impedance spectroscopy on live Caco-2 cells. **(A)** Photograph of the custom fabricated chopstick-like electrodes; **(B)** TEER values as a function of time of Caco-2 cells incubated for 32 days (inset: electric equivalent circuit model); **(C)** Bode plot of the intact Caco-2 monolayer and after 2 h and 4 h of EGTA treatment; **(D)** Bode plot of the transwell inserts with and without cells.

To validate with traditional techniques, the experimental results obtained with presented Raman microspectroscopy and the ML data analysis, TEER measurements and immunofluorescent staining were used to assess the real epithelia damage status. Trans-Epithelial Electrical Resistance (TEER) analysis was used to study the electrical properties of the cell monolayer and, in particular, to monitor the integrity of the epithelial barrier (Srinivasan et al., 2015). Impedance spectroscopy offers the possibility of a continuous and non-invasive analysis of TEER by measuring current over a range of frequencies, providing more detailed information than classic single frequency TEER measurements (Benson et al., 2013). To monitor the cellular growth and the cell-cell connection through the formation of TJs between adjacent cells, impedance spectra were recorded using custom fabricated chopstick-like electrodes (Figure 8A) after changing the cell culture medium, over a period of 32 days. The experimental impedance data were fitted using an equivalent circuit model (inset Figure 8B) representing the cellular system, where R_s_ is the resistance of the cell medium, R_(TEER)_ is the resistance of the paracellular route, C is the capacitance of both the apical and the basolateral membranes of the cells and CPE is the empirical constant phase element modeling the impedance of the electrode-medium interface. As shown in Figure 8B, TEER values increased during the cells incubation and reached 688 Ω‧cm^2^ after 32 days. These results indicate a rapid cell growth during the first 14 days after seeding, suggesting the formation of an intact cell monolayer, after which the TEER values increase very slowly. Impedance measurements were also performed on the Caco-2 cell layer after EGTA treatment to assess its effect on the TJs and consequently on the epithelial permeability. EGTA solution (5 mM) was added to the apical compartment of the Transwell insert and the integrity of the cell barrier was electrically monitored for 4 h. The Bode plot of the intact cell monolayer and after 2 h and 4 h of EGTA exposure is shown in Figure 8C. At high frequencies a resistive behavior is dominant, whereas at low frequencies the system is dominated by a capacitive behavior. The resistance (TEER) and the capacitance of the epithelium contribute mainly at mid-frequencies of the frequency spectrum with impedance magnitude and phase shown as typical of a parallel R-C circuit (Figures 8C, D). The presence of an intact cell layer (Figure 8C, blue line) determines an increase in the impedance magnitude due to the epithelial barrier resistance and a peak in the phase plot representing the capacitive contribution of the cell layer. EGTA treatment causes a gradual flattening of the magnitude and phase plots, indicating a gradual disruption of the integrity of the cell barrier. TEER values decrease from values of 700 ± 26 Ω‧cm^2^ of the intact epithelium to values of 328.9 ± 9.1 Ω‧cm^2^ and 197.9 ± 4.5 Ω‧cm^2^ after 2 h and 4 h of EGTA treatment, respectively.

These data confirm that the Raman-based ML algorithm successfully classifies *versus* the 3 different output classes (intact epithelium, 2 h and 4 h treatment) that are related to 3 different values of TEER parameters. The main advantage of the present Machine Learning-supported microspectroscopy Raman technique resides on the contactless method of measurement, avoiding any electrodes insertion into an OoC device of transwell platform.

**FIGURE 9:**
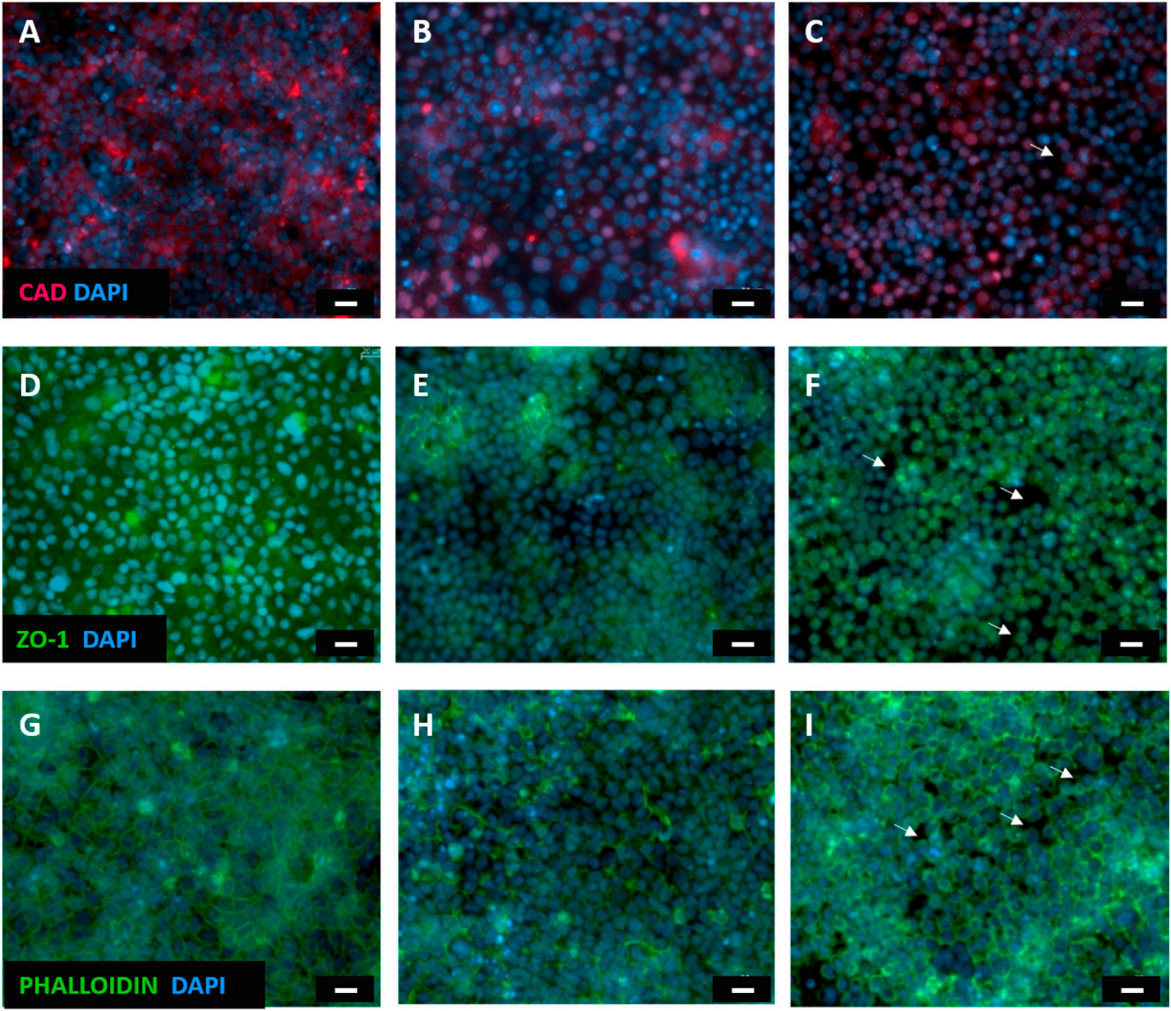
Fluorescent microscopy images of Caco-2 epithelial cell layer. Caco-2 monolayer stained with E-cadherin monoclonal antibody**(A)** ZO-1 monoclonal antibodies**(D)** and phalloidin**(G)** before any treatment with EGTA; E-cadherin **(B)**, ZO-1 **(E)** and actin microfilaments **(H)** staining 2 h after EGTA exposure; the tight junction proteins E-cadherin **(C)**, ZO-1 **(F)** and actin **(I)** staining of Caco-2 cells after a 4 h EGTA treatment. White arrows indicate cells detachment due to the epithelial injury. The nucleus was stained with DAPI (blue). Scale bar = 20 μm.

In order to complete the experimental assessment on the TJ functionality vs. the EGTA treatments, the immunofluorescent staining of intercellular junctions was carried out, following the TEER tests. The TJs are essential adhesive proteins of contact between neighboring epithelial cells; they comprise different proteins and among them, E-cadherin and ZO-1 are the major components. Therefore, it was observed the fluorescence related to these transmembrane proteins, whereas actin filaments were visualized by means of phalloidin staining. Experimental results show marked E-cadherin and ZO-1 (Figures 9A, D) as well as a homogeneous staining of actin microfilaments with phalloidin (Figure 9G) before any treatment with EGTA. After 2 h of EGTA exposure, a slightly reduced fluorescent signal related to adherent proteins and actin, was observed, thus indicating that the Caco-2 monolayer was compromised (Figures 9B, E, H). Finally, after 4 h of EGTA treatment the altered localization fluorescent staining of transmembrane proteins, the shrinkage of cell morphology and cytoskeletal elements, suggested a loss of cell adherence (Figures 9C, F, I). Indeed, the Caco-2 monolayer shows the presence of “holes” (white arrows in Figures 9C, F, I) as a consequence of the detachment of the cells induced by the 4 h EGTA treatment; these findings confirm that the models slightly suffer in discriminating between damaged epithelium and cell-free membranes due to the similarity of these two classes, likely due to the significant epithelial injury induced by a 4 h EGTA treatment and the consequent exposure of cell-free membranes. The dark background (PET membrane) visible in Figure 9 between cells suggests that Raman analysis on those points collects an appreciable signal coming from the blank substrate underneath, missing the Raman tight junctions fingerprint (Hu et al., 2013).

## 4 Conclusion

A study on Raman microspectroscopy as a marker-independent technique to assess epithelial integrity in cell cultures and Organ-on-Chip devices was presented. Multi-points, intercellular-localized measurements on Caco-2 cells monolayer using a Raman microspectroscopy were performed to detect and classify different physiological/pathological conditions in a non-invasive way. Afterwards, a data processing chain and related supervised Machine Learning algorithm (ML) was designed and trained to classifying the Raman measurements in four epithelium status classes (intact epithelium, 2 h, 4 h, empty membrane), experimenting a dual approach in dataset splitting and moving towards a pre-clinical strengthening of the whole process. The ML algorithm successfully classifies the different epithelia damage status with the Quadratic SVM classifier, reaching an accuracy of 91.9% with only 7 features, opening the possibility to adopt low-cost hardware for computational tasks.

Moreover, to further support results from the Raman microspectroscopy and the complex data analysis, TEER and immunofluorescent staining were used to assess the epithelial condition, confirming the validity and consistency of the method. Our findings confirmed that this approach represents a promising tool for a non-invasive and non-destructive characterization of cells and biological barriers in organoids platforms with applications in cytology diagnostics, tumor progression or drug efficacy analysis. Future prospects for improving Raman spectroscopy in biological tissues investigations include the development of spectroscopy-compatible OoC devices for more standardized and reproducible measurements through a precise experimental control. Additionally, biocompatible micro/nanostructures substrates can enhance the Raman signal for a more sensitive analysis. Finally, a large number of measurements coupled with automatic positioning systems will be mandatory for the development of more robust predictive models, enhancing the technique’s reliability.

## Data Availability

The original contributions presented in the study are included in the article/supplementary material, further inquiries can be directed to the corresponding author.
